# Uptake Transporters at the Blood–Brain Barrier and Their Role in Brain Drug Disposition

**DOI:** 10.3390/pharmaceutics15102473

**Published:** 2023-10-16

**Authors:** Md Masud Parvez, Armin Sadighi, Yeseul Ahn, Steve F. Keller, Julius O. Enoru

**Affiliations:** 1 Department of Quantitative, Translational & ADME Sciences (QTAS), AbbVie Biotherapeutics, San Francisco, CA 94080, USA; mdmasud.parvez@abbvie.com (M.M.P.);; 2 Department of Pharmaceutical Sciences, Jerry H. Hodge School of Pharmacy, Texas Tech University Health Sciences Center, 1300 S Coulter St., Amarillo, TX 79106, USA; 3 Center for Blood-Brain Barrier Research, Jerry H. Hodge School of Pharmacy, Texas Tech University Health Sciences Center, Amarillo, TX 79106, USA

**Keywords:** uptake transporters, blood–brain barrier, pharmacokinetics, CNS drug delivery

## Abstract

Uptake drug transporters play a significant role in the pharmacokinetic of drugs within the brain, facilitating their entry into the central nervous system (CNS). Understanding brain drug disposition is always challenging, especially with respect to preclinical to clinical translation. These transporters are members of the solute carrier (SLC) superfamily, which includes organic anion transporter polypeptides (OATPs), organic anion transporters (OATs), organic cation transporters (OCTs), and amino acid transporters. In this systematic review, we provide an overview of the current knowledge of uptake drug transporters in the brain and their contribution to drug disposition. Here, we also assemble currently available proteomics-based expression levels of uptake transporters in the human brain and their application in translational drug development. Proteomics data suggest that in association with efflux transporters, uptake drug transporters present at the BBB play a significant role in brain drug disposition. It is noteworthy that a significant level of species differences in uptake drug transporters activity exists, and this may contribute toward a disconnect in inter-species scaling. Taken together, uptake drug transporters at the BBB could play a significant role in pharmacokinetics (PK) and pharmacodynamics (PD). Continuous research is crucial for advancing our understanding of active uptake across the BBB.

## 1. Introduction

Membrane transporters are expressed in several organs and play a significant role in pharmacokinetic (PK) drug disposition, pharmacodynamics (PD), and clinical drug–drug interactions [1]. The blood–brain barrier (BBB) always challenges the permeation of drugs into the brain, especially central nervous system (CNS)-targeted therapeutics [2,3]. Properties like unbound tissue partition coefficient (Kp,u) or, more specifically, the unbound brain-to-plasma drug concentration ratio (*K*_*p*,*uu*,*brain*_) of a drug or chemical could be key determinant factors in drug penetration via BBB [4]. It is well established that drug transporters present at BBB could modulate drug delivery into the brain. To date, several in vitro, in vivo, and clinical studies have been conducted primarily to understand the role of efflux transporters in brain drug exposure. For example, brain exposure of raltegravir, an antiretroviral drug, was found to be strongly associated with p-glycoprotein (P-gp) and breast cancer-resistant protein (BCRP), with P-gp inhibitor PSC833 and BCRP inhibitor Ko143 significantly increasing raltegravir accumulation in human cerebral microvessel endothelial (hCMEC/D3) and mouse Sertoli TM4 cells [5]. On the contrary, similar investigations of the role of uptake transporters in brain drug disposition are poorly reported. However, a number of reports have demonstrated a solute carrier (SLC) family transporter expression in human microvessels, namely organic anion transporter polypeptide (OATP)-1B1, -1B3, -2B1, -1A2, organic cation transporter (OCT), organic anion transporter (OAT), equilibrative nucleoside transporters (ENTs), concentrative nucleoside transporter (CNT), monocarboxylate transporters (MCTs), L-type amino acid transporter (LAT), and multidrug and toxin extrusion transporters (MATEs) [6,7,8,9,10]. There are also examples of clinical drugs that are well-known substrates of these uptake transporters, such as erythromycin, fexofenadine, imatinib, levofloxacin, methotrexate, pitavastatin, saquinavir for OATP1A2 [11,12,13,14,15,16], and atorvastatin, benzylpenicillin, bosentan, fexofenadine, glibenclamide, and rosuvastatin that are substrates of OATP2B1 [17,18,19,20,21,22]. Several studies using these compounds have suggested their potential contribution in PK and drug–drug interactions (DDIs), leading to speculations regarding their brain distribution as well. For example, OATP1A2-mediated DDIs were demonstrated using fexofenadine as a substrate for this transporter and the exposure (AUC) of fexofenadine was reduced by 25% and 40–70% due to flavonoids naringin and grapefruit juice (OATP1A2 inhibitor), respectively [23,24,25]. OATP1A2 has also been suggested to play a significant role in male hormone dehydroepiandrosterone sulfate (DHEAS) uptake into the brain and liver [23]. The coadministration of fexofenadine (a known substrate of P-gp), with terfenadine, increased brain penetration in mice by 25–27-fold, indicating that transporter-mediated disposition could be a key mechanism for fexofenadine brain exposure [26]. Despite the examples available in the literature, there is still a gap in quantifying the relative contribution of individual uptake transporters and their role in CNS exposure, efficacy, and safety. It should be noted that the clinical relevance of these uptake transporters is not limited to CNS-targeted therapeutic drugs. These uptake transporters also play a significant role in the PK and PD of non-CNS-targeted drugs such as statins [26,27,28,29].

Thus, understanding the mechanism and extent of drug penetration into the brain remains an unmet need in drug discovery and development. In this review, we summarize the current knowledge of the tools available, protein abundance, and clinical drug substrates of uptake transporters to facilitate our understanding and propose a roadmap on the significance of uptake transporters’ contribution in brain drug disposition.

## 2. Study Highlights

We performed a comprehensive systematic review of publicly available literature data in the following areas: expression and tissue localization of uptake transporters in human, in vitro study models (cell line and rodent), in silico models, species differences, clinical PK/PD, and brain toxicity of therapeutic drugs. To capture available data, a through data mining was performed in the public domain (PubMed, Google Scholar, University of Washington DIDBW, Washington, DC, USA) by using key words (brain drug disposition, transporters in brain, transporter proteomics in brain, inter-species differences in brain drug disposition, drug-induced brain toxicity, pharmacokinetics in brain, blood–brain barrier, drug–drug interactions in brain). We included the reported studies in this review, considering study details, data quality, the concomitant use of the target drugs, pathophysiological conditions, protein quantification, and plausible data interpretation with minimal limitations. 

## 3. Localization, Functions, and Expression of the Drug Uptake Transporters in the Brain

**Figure 1:**
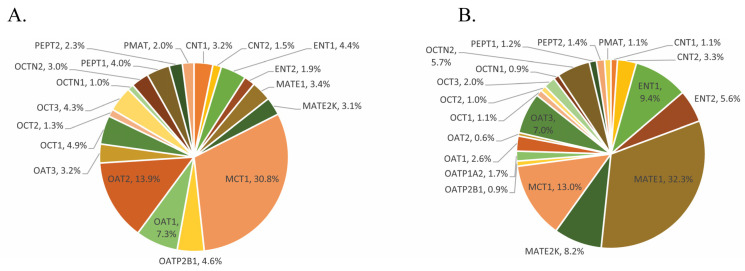
Uptake transporter proteomics in human brain cells. A summary of the literature reported proteomics-based quantitative abundance of clinically relevant major drug uptake transporters in human brain microvessels (**A**) and choroid plexus (**B**). Data represent an average percent of transporter protein absolute abundance (pmol/mg protein), summarized in Table 1. CNTs: concentrative nucleoside transporters; ENTs: equilibrative nucleoside transporters; MCTs: monocarboxylate transporters; OATPs: organic anion transporter polypeptides; PMAT: plasma membrane monoamine transporter; LATs: L-type amino acid transporters; OATs: organic anion transporters; OCTs: organic cation transporters; MATE: multidrug and toxic compound extrusion; PEPTs: peptide transporters. Graphical illustrations were made using Microsoft excel and Adobe IllustratorCC.

Results from our literature review showed that a large variety of uptake transporters are localized and significantly expressed at the BBB across species (Figure 1). There are very few studies that have demonstrated in vivo brain exposures in humans due to the difficulty in obtaining samples and a lack of in vitro-to-in vivo extrapolation (IVIVE) tools. Following recent advances in LC-MS/MS technology, the quantification of uptake transporter protein abundance is currently performed using LC-MS/MS-based proteomics. This enables translation via scaling or PBPK modeling and allows for estimations of their relative contribution in brain drug exposure. The human OATP family consists of 11 members, of which OATP1A2, 1B1, 1B3, and 2B1 play an important role in drug disposition and pharmacokinetics. Among these, OATP1A2 and OAT2B1 are highly expressed at the BBB and play a significant role in drug uptake into the brain. These OATP isoforms, namely OATP1A2, Oatp1a4, and OATP2B1 are expressed at the endothelial membrane, through which substrates enter the brain [30]. On the other hand, P-gp, BCRP, MRP1, MRP4, MRP5, and MRP2 are expressed at the luminal side that pumps substances out from the intracellular space [30]. Other uptake family transporters such as OCT1, OCT2, and OCTN2 are mainly expressed on the luminal side of brain microvessel endothelial cells and play a role in substrates’ uptake into the brain [31]. OCTN2 and OCTN3 were reported to be expressed in rodent cell lines that uptake substrates into the brain that align with membrane potential and the proton gradient. However, these transporters need further investigation to confirm their localization in microvessels and choroid plexus epithelial cells in order to understand their contribution in CNS therapeutics [32,33,34]. MCT transporters are widely expressed in rat, mouse, or human brain endothelial cells, ependymocytes, and astrocytes, playing an important role in uptake into the brain [35]. MCTs facilitate lactate and monocarboxylates transport and cellular metabolism in a proton-dependent manner [36]. Apart from endogenous substances, MCTs also transport therapeutic drugs like atorvastatin and valproic acid [37]. Another uptake transporter at the BBB is LAT1, which is localized in both the apical and basolateral membrane of the brain capillary endothelial cells [38]. LAT1 is composed of a total 15 members and categorized into two subgroups: cationic amino acid transporters and LAT heterodimeric amino acid transporters [38]. LAT1 forms a heterodimeric amino acid transporter interacting with the glycoprotein CD98 to exert an uptake of a broad range of amino acids (tryptophan, phenylalanine, leucine, and histidine), prodrugs, and thyroid hormones T3 and T4 [39,40,41].

BBB limits the brain penetration of therapeutics and reduces their efficacy in the treatment of brain malignancies and other CNS disorders [42]. Targeting uptake transporters to overcome the tightly integrated BBB and efficiently improve drug delivery to the site of action is desirable [43]. Thus, an accurate estimation of uptake transporters’ absolute expression at the BBB will provide more insight into our mechanistic understanding of drug penetration [10]. This will help in predicting the bioavailability and disposition of current and future therapeutics [44]. Quantitative targeted absolute proteomics technique (QTAP) analyzed via targeted LC-MS/MS has been used to identify and quantify the expression of uptake transporters in the human brain. Human choroid plexus [9], human brain microvessels (BMVs) [8], and hCMEC/D3 cells [7] have been used for quantitative targeted absolute proteomics of human brain tissues and cells. Uchida and co-workers [9] stated that four selected/multiple reaction monitoring (SRM/MRM) transitions have been optimized for the identification and quantification of target peptide. To achieve accurate protein quantification with coefficients of variation (CV) of <20.0%, three or four positive peaks must be determined. When no positive peak is detected or only one or two SRM/MRM transitions occur for a specific protein, the protein expression is expressed as under the limit of quantification (ULQ). In other words, the sensitivity of the third most transition determines the LQ (fmol/μg protein). The absolute quantification of uptake transporters in the human brain (tissue and cells) has been reviewed here with more emphasis on FDA-recommended uptake transporters. Table 1 summarizes the list of uptake transporters quantified via targeted LC-MS/MS proteomics with their absolute quantitation in different regions of the human brain and plasma membrane fraction of hCMEC/D3 cells. 

OATPs are responsible for the uptake of a wide range of structurally diverse substrates, from endogenous substances like steroid hormones and bile acids to statins and chemotherapeutics. The first attempt to find OATPs in human brain was accomplished by Kullak-Ublick and co-workers in search of a dehydroepiandrosterone sulfate (DHEAS) uptake protein [23]. In 1998, they provided the very first proof of the presence of OATPs in the brain based on the Northern blotting technique. Subsequently, the presence of OATP1A2 in the human BBB endothelial cells and brain capillaries was confirmed via immunohistochemical staining and Western blotting [45,46]. The expression and localization of six OATPs (i.e., OATP1A2, OATP1B1, OATP1B3, OATP1C1, OATP2B1, and OATP4A1) have been proven by Bronger et al. [47] in the endothelial cells of human gliomas. The localization of OATP1A2 and OATP2B1 at the BBB and the blood–tumor barrier has been identified at mRNA level and protein immunoblotting [47]. Uchida and co-workers [9] have identified and quantified OATP1A2 and OATP2B1 in a plasma membrane fraction of human choroid plexus (0.45 and 0.24 fmol/μg < ULQ) through a targeted LC-MS/MS approach. Moreover, Al-Majdoub and colleagues [6] quantified OATP2B1 as 0.40 to 0.48 fmol/μg total protein, using the same approach. Among the OATP family, OATP1B1 and OATP1B3 are recommended by the FDA for clinical DDI liability. Uchida and co-workers [9] have identified OATP1B1 and 1B3 as 0.30 and 0.62 fmol/μg, respectively, in a human choroid plexus; however, they were considered as ULQ (Table 1).

The SLC superfamily transporters, including OATs, OCTs, and novel organic cation transporters (OCTNs), are primarily responsible for the uptake of circulating solutes from blood to the brain [48]. Billington and co-workers have identified the inter-individual and inter-regional variability of drug transporters expression in the human brain using quantitative targeted proteomics [49]. According to their study, the abundance of OAT3, OCT1/2, and OCTN1/2 were estimated as below the limit of quantification. In another attempt, Al-Majdoub et al. [6] quantified the expression of OAT1, 2, 3, and 7 as 0.48, 7.9, 0.27, and 0.51 pmol/mg of the total protein in human BMVs, respectively. In addition, Uchida and co-workers [9] quantified OAT3 as 1.87 pmol/mg of plasma membrane fraction in human choroid plexus. Giacomini’s group has identified the presence of OCT3 in human BMVs from two donors using the immunohistochemistry method [32]. This finding was supported by Al-Majdoub and co-workers, who identified the exact expression level of OCT3 as 0.62 ± 0.08 pmol/mg total protein in human BMVs [6]. In a more recent study, the absolute expression of OCT3 in human BMVs was estimated as 0.15 ± 0.056 fmol/μg of total protein using targeted LC-MS/MS-based proteomics [49]. This study has shown that OCT3 is the most highly expressed OCT in human BBB, while OCT1 and 2 are not detectable at the protein level. Multidrug and toxin extrusion protein 1 (MATE1) and MATE2-K have been found in the plasma membrane of human choroid plexus as 8.61 ± 0.63 and 2.19 fmol/μg protein (ULQ), respectively [9]. Moreover, MATE1 and MATE2-K have been quantified as ULQ as 0.33 and 0.29 fmol/μg protein in human BMVs (Table 1). 

Other than FDA-recommended uptake transporters in the human brain, there are other uptake transporters essential for brain physiology [50]; LAT1(SLC 7A5/SLC2A3), which is responsible for the uptake of large neutral amino acids, thyroid hormones, and medicines, is one of them [51,52]. LAT1 is a light-chain amino acid uptake transporter connected by a disulfide bond to the heavy chain 4F2 cell-surface antigen heavy chain (4F2hc). The formation of the LAT1/4F2hc heteromeric complex is essential for the stabilization and localization of LAT1 in the BBB membrane [51,52]. Moreover, the overexpression of LAT1 in some tumor cells has been identified as a promising target in cancer therapy [51,53]. The absolute expression of LAT1 was quantified to be between 0.43 and 0.71 pmol/mg total plasma membrane protein fraction in healthy human BMVs [6,8]. Ohtsuki and co-workers have found 4F2hc, MCT1, ENT1 uptake transporters in both hCMEC/D3 cells and human BMVs [7]. MCTs play a crucial role in cellular metabolism by facilitating the transport of endogenous monocarboxylates like lactate into and out of brain cells [37]. ENTs are bidirectional, sodium-independent transporters involved in the inward and outward transport of nucleosides [54]. The expression levels of 4F2hc, MCT1, and ENT1 in the plasma membrane fraction of hCMEC/D3 cells were reported as 1.90 ± 0.23, 1.87 ± 0.22, and 5.94 ± 0.35 fmol/μg of protein, respectively (Table 1) [7]. The expression of ENT1 in human BMVs was estimated from 0.27 ± 0.1 [6] to 0.57 ± 0.13 fmol/μg protein [8]. The absolute expression of ENT1 was reported as 2.49 ± 0.12 fmol/μg protein in the plasma membrane fraction of human choroid plexus [9], while the expression of ENT2 in human BMVs and choroid plexus was ULQ (Table 1) [7,8].

### 3.1. Models Used to Study Uptake Transporters-Mediated Brain Drug Disposition

**Figure 2:**
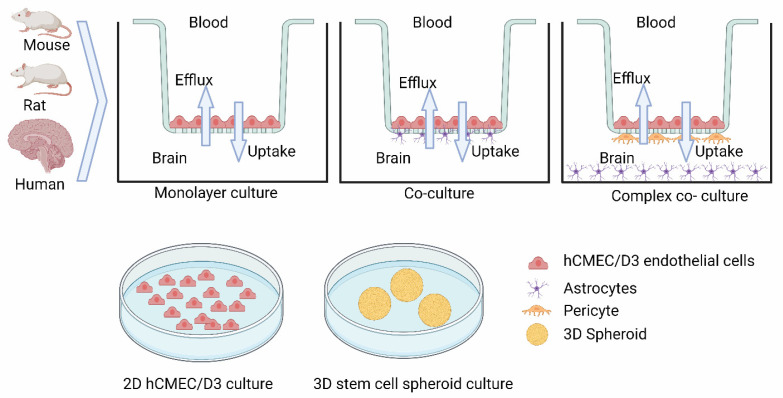
In vitro blood–brain barrier models for drug transporter studies. Graphical presentation of currently available in vitro models for drug transporter studies in different species (mouse, rat, human). In vitro models include brain endothelial microvascular cell-based culture for permeability into the brain (Kp,uu) using monolayer, co-culture with astrocytes; triple culture with pericytes and astrocytes (upper panel). Uptake transporter functional assay in vitro models showing 2D model with human microvascular endothelial cells (hCMEC/D3) and 3D culture with stem cell-derived spheroids. Graphical illustrations were made with BioRender and Adobe IllustratorCC.

The BBB is a complex system that is made up of low-permeable brain capillary endothelial cells. This leads to a reduced transcytosis and tight junctions, resulting in very low paracellular transport [55]. Thus, many endogenous and exogenous compounds (e.g., drugs) need active transport processes to facilitate distribution in the brain [41]. In vitro brain cell models have been in use for over 50 years [56]. However, creating a BBB-like in vitro system is quite challenging. So far, several in vitro cell line models have been proposed in different species. Examples include: (i) immortalized and primary mouse brain endothelial cells, (ii) mono-culture rat brain capillary endothelial cells (BCEC), (iii) co-culture rat BCEC, (iv) triple-culture rat BCEC, (v) astrocytes co-culture with BCEC bovine cells, (vi) porcine monoculture cells with porcine brain endothelial cells (PBEC), (vii) human endothelial cells (hCMEC/D3), (viii) human BBB model with pluripotent stem cells (hPSCs), (ix) cord blood-derived endothelial progenitor cells, and (x) an hPSC-derived 3D spheroid system (Figure 2, an updated graphical presentation from HC Helms et al. [56]). Apart from these in vitro models, in vivo models have also been used, and include mouse, rat, monkey, and dog. Also very recently, proteomics-based IVIVE and physiologically based pharmacokinetic model (PBPK) data were published [41,42,57]. The isolation of these brain cell lines started since the 1970s and tremendous progress has been made toward optimizing isolation methods, as well as culture and phenotyping for transporter studies [27,58,59,60,61]. Since the establishment of monolayer culture models, several studies have shown progress on tight junction formation to study permeability and efflux transporters contribution. The majority of these studies used endothelial cells with astrocytes or pericytes [58,62,63,64,65]. Each of these models has its own specific advantage over the other. For example, triple-culture rat BCEC consists of BCEC cells with astrocyte/pericyte that facilitates the formation of monolayers having spindle shapes, that are known to express occludin and increase trans-epithelial electric resistance (TEER) [66,67]. The primary mouse BCEC–astrocytes co-culture system shows prominent tight junction (TEER up to 1000 Ω·cm^2^); however, uptake transporter functions have not yet been characterized in this model [68,69,70,71]. Rat primary endothelial BCEC cells were characterized for P-gp, BCRP, MRP-1, and MRP1 functions. Rat BCEC cells express other uptake transporters such as Glut-1, LAT1, and PMAT [72,73,74]. Also, published results suggest that serum-free monolayer culture conditions are more sensitive to Glut-1-like uptake transporter downregulation than the efflux transporter expression [75,76]. hCMEC/D3 cell line first developed in 2005 represents a stable, easy growing, and maintenance line, with high translational capacity similar to brain microvascular endothelial cells in the BBB [77]. The hCMEC/D3 cells are derived from the hTERT/SV40-immortalized cloned cells from human temporal lobe microvessels isolated from an epileptic patient. On the other hand, hCMEC/D3 cells show a low TEER value, suggesting that an improvement in culture conditions is warranted. This led to the evaluation of a co-culture system with astrocytes and pericytes [78,79]. So far, over 140 uptake transporters (SLC family) have been identified in hCMEC/D3 cell lines, including Glut-1, LAT-1, MCT, and OATPs [5,77,80,81]. The hCMEC/D3 model has also been used for the discovery and development of antihistaminic drug candidates that need to reach the CNS [27]. A summary of the currently available opportunistic in vitro cell-based models is shown in Figure 2. 

### 3.2. Species Differences in Uptake Transporter Activity and Drug–Drug Interactions in Brain

Studies have identified a significant level of inter-species differences in brain drug dispositions. These species differences are known to be caused by physiological changes, especially a differential expression of the transporters and enzymes among species (i.e., mouse, rat, dog, monkey, and human). Table 2 summarizes a few examples of uptake transporters involved in rat, mouse, monkey, dog, and human brain uptake. As shown in this table, memantine brain uptake was 84.59 ± 9.73 pmol/mg brain tissue in control male rats, while it decreased by 36% in the presence of OCT1/2 inhibitor (cimetidine, 25 μM). In situ brain perfusion results showed that cimetidine reduced the brain uptake of memantine in ipsilateral and contralateral cerebral cortices from both MCAO animals and sham-operated controls [82]. In another example, the increased functional expression of Oatp1A4 in the presence of bone morphogenetic protein-9 (BMP-9) resulted in the higher extent of atorvastatin, pravastatin, and rosuvastatin brain absorption. Briefly, the pharmacological inhibition of the ALK1 receptor with LDN193189 resulted in an attenuation of the increased brain exposure observed in the presence of BMP-9 only for all three statin drugs [83]. In situ brain perfusion studies in control rats confirmed the specificity of Oatp-mediated transport with a reduced whole-brain uptake of all three statin drugs in the presence of fexofenadine (FEX), an Oatp inhibitor [83]. Similarly, SHY-01 showed higher brain uptake levels in rats than metformin hydrochloride at 1 h (0.32 ± 0.023 vs. 0.19 ± 0.032 μg/g; *p* = 0.005) and 2 h (0.25 ± 0.032 vs. 0.11 ± 0.012 μg/g; *p* = 0.002) after oral administration [84]. In rat, digoxin (2 mg/kg, i.v.) showed low brain penetration (K_P,AUC,Brain_ = ~0.07); however, when treated with both elacridar (P-gp inhibitor) and rifampicin (OATP inhibitor), the K_P,AUC,Brain_ for digoxin increased by 6-fold, whereas the K_P,AUC,Brain_ reduced by 2-fold (0.89 to 0.42) in the presence of rifampicin. Also, digoxin concentration in CSF (K_P,AUC,CSF_) increased (~4-fold) after treatment with rifampicin, suggesting a CSF-to-blood direction of an uptake transporter such as Oatp1a4, which is a homolog of human OATP1A2 [85]. In another rat study, rifampicin treatment resulted in a drastic reduction in glyburide liver uptake with a decrease in liver exposure. As a consequence of a reduced liver uptake, the concentrations of glyburide in the systemic circulation (AUC_blood_) increased, which led to an increased tissue exposure in the brain. The brain PET (positron emission tomography) scan images showed that the brain uptake of glyburide is negligible, although OATP2B1 and OATP1A2 are expressed at the BBB. The brain penetration of glyburide is restricted by efflux transporters [86]. In a baboon monkey study, glyburide showed low brain penetration (SUV_max_ = 0.6); however, the AUC_brain_/AUC_plasma_ ratio was not affected by either rifampicin, cyclosporine A, or pantoprazole treatment. For in situ brain perfusion using wild-type mice, the intrinsic brain transport rate of glyburide, K_in_, was 0.50 ± 0.11 μL/g/s (∼1.2% of the perfused glyburide). The glyburide brain uptake did not significantly change after co-perfusion with rifampicin (inhibitor of OATPs), suggesting that OATP transporters play only a minor role in the tubular excretion or reabsorption of glyburide and its metabolites [87]. A rat in situ perfusion model study for OATP substrates pitavastatin, rosuvastatin, pravastatin, and taurocholate show a significantly reduced (>2-fold) brain uptake in Oatp1a4(−/−) mice compared to wild-type mice [88]. Furthermore, BMP-9 treatment increased the expression level of Oatp1a4 in rat brain and enhanced the brain delivery of atorvastatin and pravastatin. This increased up to 93% of brain uptake than controls (no BMP-9 treatment). The brain accumulation of [^3^H]taurocholate reduced down to 69% when BMP-9 was pre-administered in presence and absence of Oatp inhibitor (i.e., estrone-3-sulphate, fexofenadine, or BSP), suggesting the significant role of OATP-mediated brain uptake in rat [89]. In a study in dogs, the proteomics-based protein abundance was found to be below LOQ for the SLC transporters OAT3, OCTs, and OATPs-1A2 and -2B1 in brain capillaries. However, in choroid plexus, OATP1A2 was detectable (Table 2) and an OCT3 and P-gp substrate quinidine resulted in a lower K_p,uu,CSF_ than the K_p,uu,brain_ (Table 2), while K_p,uu,CSF_ of dantrolene (OAT2 and BCRP substrate) was 8-fold higher than K_p,uu,brain_, suggesting the differential expression of uptake transporters in microvessels vs. choroid plexus (Table 2) Brau [90]. The difference in brain and CSF concentration of dantrolene in dogs is considerably higher than in rats [91]. The study findings were explained by P-gp and BCRP expression; however, quinidine is reported to be a substrate of OCT3 [92], and dantrolene is a substrate of OAT2 and OAT3 [93]. The species difference in the brain exposure of these two efflux/uptake transporter substrates might leverage the uptake/efflux relative ratio with expressional difference. There is an interesting finding in rat, the Oatp1a4 protein expression in brain microvessels, showing an enhanced Oatp1a4 activity upon subjection to peripheral pain. Indeed, during peripheral inflammatory pain induced by carrageenan injection, the brain accumulation of Oatp substrate taurocholate significantly increased. The in situ perfusion using rat brains showed more than a 2-fold reduction in the uptake of taurocholate when treated with various Oatp transport inhibitors, E3S, digoxin, and fexofenadine, but not with BSP treatment in both healthy and carrageenan-induced inflammatory pain models [94]. In another classic example of the OAT uptake transporter activity in rats, cefadroxil (OAT, MRP, and OATP substrate) levels were observed in blood, brain ECF, and CSF with the co-administration of probenecid. OATs and MRPs are expressed at BCSFB to pump out cefadroxil from CSF to blood, so the inhibition of these transporters by probenecid increased the K_p,uu,CSF_ of cefadroxil (Table 3). The brain slice experiments demonstrated that PEPT2 inhibition by Ala–Ala and GlySar significantly reduced the V_u,brain_ of cefadroxil, indicating PEPT2 is involved in the uptake of cefadroxil into brain cells. In contrast, the presence of probenecid increased the brain cell uptake of cefadroxil and the mechanism by which this happens is still poorly understood. However, it was speculated and proposed that probenecid may be blocking MRP efflux [95]. In an in vitro study, human (hCMEC/D3) and mouse (Bend.3) cell lines were used in an uptake study of pentamidine with or without the known OCT inhibitors, amantadine (OCT1 and OCT2 inhibitor), prazosin (OCT1 and OCT3), corticosterone (OCT3 inhibitor), and N-methylnicotinamide (OCT2 inhibitor). Amantadine significantly decreased the accumulation of pentamidine in both cell lines; however, prazosin only had significant effects on hCMEC/D3 cells. This could be due to the toxicity of prazosin to the Bend.3 cells, resulting in a leaky BBB model. The specific OCT2 inhibitor, N-methylnicotinamide, did not show any significant changes in intracellular uptake. Taken together, it was concluded that OCT1 is the key transporter present in both cell lines responsible for the uptake of pentamidine [96]. An invitro study found that 5% of grapefruit juice reduced estrone-3-sulfate (a substrate of OATP2B1) uptake by 80% [18]. OCT1 and OCT2 substrate N-methyl-4-phenyl-1,2,3,6-tetrahydropyridine (MPTP) transport was inhibited by the coadministration of amantadine in rats. The study showed that MPTP concentration in extracellular fluid reduced by 60% and 85% in rats and mice, respectively, suggesting a role for these uptake transporters in brain drug disposition. This observation was consistent among rats and mice [31].

### 3.3. Significance of Uptake Transporters in Brain Drug Disposition

Table 3 summarizes the reported *K*_*p*,*uu*,*brain*_ for different compounds, including dolutegravir (DTG), efavirenz (EFV), erlotinib (ERL), fexofenadine (FEX), gabapentin (GBP), lamotrigine (LMG), loperamide (LPM), methotrexate (MTX), pitavastatin (PTV), quinidine (QND), raltegravir (RLT), rifampicin (RFP), rosuvastatin (RSV), and zidovudine (ZDV). *K*_*p*,*uu*,*brain*_ is defined as the unbound brain-to-plasma drug concentration ratio calculated using Equation (1):

(1) $$K_{p,uu,brain}=\frac{f_{u,brain}\times C_{brain}}{f_{u,plasma}\times C_{plasma}}$$

where, $f_{u,brain}$ and $f_{u,plasma}$ stand for unbound fraction of drug in the brain and plasma, respectively. $C_{brain}$ and $C_{plasma}$ are the total drug concentration in brain and plasma, respectively. 

In addition to the *K*_*p*,*uu*,*brain*_ values, in vitro efflux and uptake brain transporters involvement in the disposition of drugs were retrieved from different literature studies to elucidate the probable role of transporters in brain exposure. MDR1 and BCRP are two major efflux transporters in the BBB that, along with uptake transporters OATPs, OCTs, OCTNs, OATs, LAT1, MATE 1, and MATE2K, are listed in Table 3. Among all the listed compounds in Table 3, RSV shows the highest *K*_*p*,*uu*,*brain*_ of 3.97 in rat [157]. This might be due to the involvement of low-capacity OATP2B1 and OATP1A2 uptake transporters in BBB. This observation warrants additional validation in vitro using OATP2B1 and OATP1A2 orthologs in rat. Table 3 shows LMG with the highest *K*_*p*,*uu*,*brain*_ in human at 2.8, suggesting the involvement of OCT1, OCT2, and OCT3 uptake transporters in human BBB [128]. Furthermore, the reported *K*_*p*,*uu*,*brain*_ for MTX is very low (0.006 in rat and 0.04 in monkey) with a significant level of species difference [104,125]. The reason for this low brain penetration of MTX might be due to the efflux through MDR1 and BCRP, which may counter the MTX influx through BBB.

## 4. Conclusions and Future Directions

**Figure 3:**
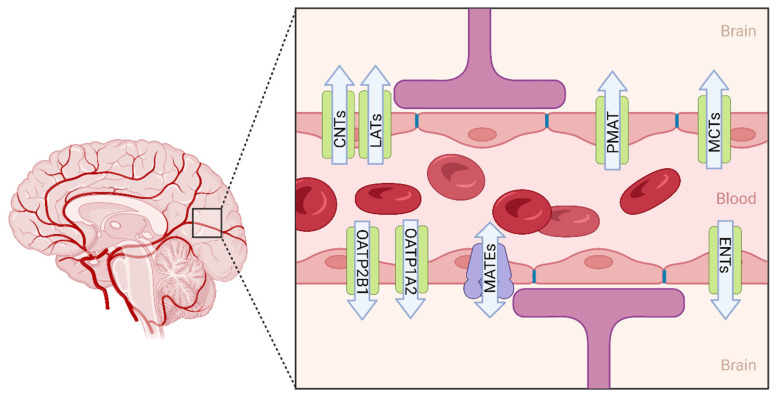
Localization of uptake transporters at the blood–brain barrier. A summary of the major drug and amino acids’ uptake transporters localization at BBB endothelial cells. The graphical localization shows the uptake transporters and their functional direction for taking drugs and amino acids through the BBB into the brain. CNTs: concentrative nucleoside transporters; ENTs: equilibrative nucleoside transporters; MCTs: monocarboxylate transporters; OATPs: organic anion transporter polypeptides; PMAT: plasma membrane monoamine transporter; LATs: L-type amino acid transporters; MATE: multidrug and toxic compound extrusion. Graphical illustrations were made with BioRender and Adobe IllustratorCC.

One major concern in the development of CNS target drugs is how the BBB affects brain exposure. CNS delivery of many compounds is greatly restricted by the BBB. To date, several strategies have been reported for its use in enhancing drug delivery to the brain. These include systemic and local routes of administration and comprise the following intranasal route, viral vectors, nanocarriers, and formulations (i.e., nanoparticles) [161]. Another attractive approach to brain drug delivery has been the linking of drugs to amino acids that actively cross the BBB, e.g., a methotrexate (MTX)–lysine conjugate enhances MTX brain uptake through the endogenous transporter system of lysine [162]. In this manuscript, we summarize the up to date information on uptake transporters’ involvement in brain disposition, citing specific studies and examples of the role of uptake transporters. While a lot more information exists on the role of efflux transporters in brain drug disposition, the equivalent information for uptake transporters at the BBB is quite sparse, and this limits the success of research on CNS-targeted drugs. Assembling the brain localization (Figure 3) and functions, proteomic expression (Table 1), species differences (Table 2) in uptake transporter activity, and the currently available in vitro models (Figure 2) not only highlights the tools available, but also sheds light on the potential preclinical to clinical translation and evaluation of DDI potential. However, the clinical relevance of these transporter contributions remains to be elucidated in a broader sense. For example, the OCT1/2 inhibitor cimetidine decreased memantine brain concentration by 37% in rat [82]. On the other hand, rifampicin, an inhibitor of OATP2B1 and OATP1A2, had no effect on glyburide brain exposure in humans [86]. Therefore, with the information assembled in this review, it is clear that additional tools and investigations are needed to further our understanding of the clinical relevance of uptake transporters expressed at the BBB. Taken together, we believe that there are a few approaches that hold promise and could be helpful in investigations regarding the role of uptake transporters on CNS drug disposition. These include: (i) proteomics-based extrapolations of the fractional transport (ft) to in vivo levels, (ii) PBPK modeling and simulations to predict brain compartmental concentrations of the drugs, (iii) transgenic/knock-out animal models for use in evaluating the role of these transporters on drug brain exposure, and (iv) a 3D stem cell-based assessment of low-clearance drugs. Furthermore, it will be highly beneficial if we can explore the rate-limiting steps involved in CNS drug exposure by investigating the interplay between uptake and efflux transport at the BBB. 

Indeed, future successes in the development of CNS-targeted drugs will depend on improvements in our understanding of BBB uptake transport mechanisms. Research should focus on investigations of the relative contributions of different uptake processes and their role in human brain drug disposition. The identification of brain uptake transporter-specific inhibitors will enhance our understanding of how large (>450 Daltons) and water-soluble drug molecules enter the brain. Overall, this review improves our current knowledge of brain uptake transporters and recommends that additional investigations are warranted in this field. Information gained so far from BBB uptake transporter mechanistic studies can be applied throughout the drug development process, and will provide a better understanding of human brain drug disposition.

## Figures and Tables

**Table 1:** Uptake transporters proteomics in human blood–brain barrier.

Transporter	Gene Name	Method	Protein Expression Level		Unit	Tissue/Cell	Reference
			Value	SD			
OCT1	*SLC22A1*	Targeted LC-MS/MS	ULQ < 0.289		fmol/μg protein	Choroid plexus (Plasma membrane fraction)	[9]
OCT1	*SLC22A1*	Targeted LC-MS/MS	ULQ < 0.288		fmol/μg protein	Brain microvessels	[8]
OCT1	*SLC22A1*	Targeted LC-MS/MS	0.58	0.11	pmol/mg protein	Brain microvessels	[6]
OCT1	*SLC22A1*	Targeted LC-MS/MS	0.54	0.06	pmol/mg protein	Brain microvessels	[6]
OCT2	*SLC22A2*	Targeted LC-MS/MS	ULQ < 0.254		fmol/μg protein	Choroid plexus (Plasma membrane fraction)	[9]
OCT2	*SLC22A2*	Targeted LC-MS/MS	ULQ < 0.123		fmol/μg protein	Brain microvessels	[8]
OCT3	*SLC22A3*	Targeted LC-MS/MS	ULQ < 0.534		fmol/μg protein	Choroid plexus (Plasma membrane fraction)	[9]
OCT3	*SLC22A3*	Targeted LC-MS/MS	ULQ < 0.207		fmol/μg protein	Brain microvessels	[8]
OCT3	*SLC22A3*	Targeted LC-MS/MS	0.62	0.08	pmol/mg protein	Brain microvessels	[6]
4F2hc	*SLC3A2*	Targeted LC-MS/MS	1.42	0.28	fmol/μg protein	Choroid plexus (Plasma membrane fraction)	[9]
4F2hc	*SLC3A2*	Targeted LC-MS/MS	3.47	0.83	fmol/μg protein	Brain microvessels	[8]
4F2hc	*SLC3A2*	Targeted LC-MS/MS	1.9	0.23	fmol/μg protein	hCMEC/D3 (plasma membrane fraction)	[7]
ASBT	*SLC10A2*	Targeted LC-MS/MS	ULQ < 0.288		fmol/μg protein	Choroid plexus (Plasma membrane fraction)	[9]
ASBT	*SLC10A2*	Targeted LC-MS/MS	ULQ < 0.12		fmol/μg protein	Brain microvessels	[8]
ASCT1	*SLC1A4*	Targeted LC-MS/MS	ULQ < 0.331		fmol/μg protein	Choroid plexus (Plasma membrane fraction)	[9]
ASCT2	*SLC1A5*	Targeted LC-MS/MS	ULQ < 1.38		fmol/μg protein	Choroid plexus (Plasma membrane fraction)	[9]
ASCT2	*SLC1A5*	Targeted LC-MS/MS	ULQ < 0.142		fmol/μg protein	Brain microvessels	[8]
ATA1	*SLC38A1*	Targeted LC-MS/MS	ULQ < 1.28		fmol/μg protein	Choroid plexus (Plasma membrane fraction)	[9]
ATA1	*SLC38A1*	Targeted LC-MS/MS	ULQ < 0.175		fmol/μg protein	Brain microvessels	[8]
ATA1	*SLC38A1*	Targeted LC-MS/MS	1.57	0.06	fmol/μg protein	hCMEC/D3 (plasma membrane fraction)	[7]
ATA2	*SLC38A2*	Targeted LC-MS/MS	ULQ < 0.497		fmol/μg protein	Choroid plexus (Plasma membrane fraction)	[9]
ATA2	*SLC38A2*	Targeted LC-MS/MS	ULQ < 0.143		fmol/μg protein	Brain microvessels	[8]
ATA3	*SLC38A4*	Targeted LC-MS/MS	ULQ < 0.823		fmol/μg protein	Choroid plexus (Plasma membrane fraction)	[9]
ATA3	*SLC38A4*	Targeted LC-MS/MS	ULQ < 0.0656		fmol/μg protein	Brain microvessels	[8]
BGT1	*SLC6A12*	Targeted LC-MS/MS	ULQ < 1.95		fmol/μg protein	Choroid plexus (Plasma membrane fraction)	[9]
BGT1	*SLC6A12*	Targeted LC-MS/MS	3.16	0.94	fmol/μg protein	Brain microvessels	[8]
BOCT	*SLC22A17*	Targeted LC-MS/MS	ULQ < 0.265		fmol/μg protein	Choroid plexus (Plasma membrane fraction)	[9]
BOIT	*SLC22A17*	Targeted LC-MS/MS	ULQ < 0.503		fmol/μg protein	Brain microvessels	[8]
CAT1	*SLC7A1*	Targeted LC-MS/MS	1.13	0.18	fmol/μg protein	Brain microvessels	[8]
CAT1	*SLC7A1*	Targeted LC-MS/MS	1.22	0.15	fmol/μg protein	Choroid plexus (Plasma membrane fraction)	[9]
CNT1	*SLC28A2*	Targeted LC-MS/MS	ULQ < 0.297		fmol/μg protein	Choroid plexus (Plasma membrane fraction)	[9]
CNT1	*SLC28A1*	Targeted LC-MS/MS	ULQ < 0.308		fmol/μg protein	Brain microvessels	[8]
CNT2	*SLC28A2*	Targeted LC-MS/MS	ULQ < 0.867		fmol/μg protein	Choroid plexus (Plasma membrane fraction)	[9]
CNT2	*SLC28A2*	Targeted LC-MS/MS	ULQ < 0.141		fmol/μg protein	Brain microvessels	[8]
CNT3	*SLC6A8*	Targeted LC-MS/MS	ULQ < 0.35		fmol/μg protein	Choroid plexus (Plasma membrane fraction)	[9]
CNT3	*SLC28A3*	Targeted LC-MS/MS	ULQ < 0.552		fmol/μg protein	Brain microvessels	[8]
CRT1	*SLC6A8*	Targeted LC-MS/MS	ULQ < 0.0915		fmol/μg protein	Brain microvessels	[8]
CT2	*SLC22A16*	Targeted LC-MS/MS	ULQ < 0.122		fmol/μg protein	Brain microvessels	[8]
CTL1	*SLC44A1*	Targeted LC-MS/MS	ULQ < 0.293		fmol/μg protein	Choroid plexus (Plasma membrane fraction)	[9]
CTL2	*SLC44A2*	Targeted LC-MS/MS	ULQ < 0.383		fmol/μg protein	Choroid plexus (Plasma membrane fraction)	[9]
EAAT1	*SLC1A3*	Targeted LC-MS/MS	5.04	0.18	fmol/μg protein	Choroid plexus (Plasma membrane fraction)	[9]
EAAT3	*SLC1A1*	Targeted LC-MS/MS	ULQ < 0.256		fmol/μg protein	Brain microvessels	[8]
EEAT1	*SLC1A3*	Targeted LC-MS/MS	25.4	12.5	fmol/μg protein	Brain microvessels	[8]
ENT1	*SLC29A1*	Targeted LC-MS/MS	0.568	0.134	fmol/μg protein	Brain microvessels	[8]
ENT1	*SLC29A1*	Targeted LC-MS/MS	5.94	0.35	fmol/μg protein	hCMEC/D3 (Plasma membrane fraction)	[7]
ENT1	*SLC29A1*	Targeted LC-MS/MS	0.27	0.1	pmol/mg protein	Brain microvessels	[6]
ENT1	*SLC29A1*	Targeted LC-MS/MS	2.49	0.12	fmol/μg protein	Choroid plexus (Plasma membrane fraction)	[9]
ENT2	*SLC29A2*	Targeted LC-MS/MS	ULQ < 1.49		fmol/μg protein	Choroid plexus (Plasma membrane fraction)	[9]
ENT2	*SLC29A2*	Targeted LC-MS/MS	ULQ < 0.18		fmol/μg protein	Brain microvessels	[8]
FATP1	*SLC27A1*	Targeted LC-MS/MS	ULQ < 1.04		fmol/μg protein	Choroid plexus (Plasma membrane fraction)	[9]
FATP2	*SLC27A2*	Targeted LC-MS/MS	ULQ < 0.199		fmol/μg protein	Choroid plexus (Plasma membrane fraction)	[9]
FATP3	*SLC27A3*	Targeted LC-MS/MS	ULQ < 0.44		fmol/μg protein	Choroid plexus (Plasma membrane fraction)	[9]
FLIPT1	*SLC22A15*	Targeted LC-MS/MS	ULQ < 0.245		fmol/μg protein	Choroid plexus (Plasma membrane fraction)	[9]
FLIPT1	*SLC22A15*	Targeted LC-MS/MS	ULQ < 0.101		fmol/μg protein	Brain microvessels	[8]
GAT2	*SLC6A13*	Targeted LC-MS/MS	ULQ < 1.3		fmol/μg protein	Choroid plexus (Plasma membrane fraction)	[9]
GAT2	*SLC6A13*	Targeted LC-MS/MS	ULQ < 0.374		fmol/μg protein	Brain microvessels	[8]
GLUT2	*SLC2A2*	Targeted LC-MS/MS	ULQ < 5.85		fmol/μg protein	Choroid plexus (Plasma membrane fraction)	[9]
GLUT4	*SLC2A4*	Targeted LC-MS/MS	ULQ < 2.52		fmol/μg protein	Choroid plexus (Plasma membrane fraction)	[9]
GLUT4	*SLC2A4*	Targeted LC-MS/MS	ULQ < 0.136		fmol/μg protein	Brain microvessels	[8]
LAT1	*SLC7A5*	Targeted LC-MS/MS	ULQ < 0.76		fmol/μg protein	Choroid plexus (Plasma membrane fraction)	[9]
LAT1	*SLC7A5*	Targeted LC-MS/MS	0.431	0.091	fmol/μg protein	Brain microvessels	[8]
LAT1	*SLC7A5*	Targeted LC-MS/MS	0.59	0.15	pmol/mg protein	Brain microvessels	[6]
LAT1	*SLC7A5*	Targeted LC-MS/MS	0.71	0.25	pmol/mg protein	Brain microvessels	[6]
LAT2	*SLC7A6*	Targeted LC-MS/MS	ULQ < 0.059		fmol/μg protein	Brain microvessels	[8]
LAT2	*SLC7A6*	Targeted LC-MS/MS	ULQ < 2.08		fmol/μg protein	Choroid plexus (Plasma membrane fraction)	[9]
MATE1	*SLC47A1*	Targeted LC-MS/MS	ULQ < 0.33		fmol/μg protein	Brain microvessels	[8]
MATE1	*SLC47A2*	Targeted LC-MS/MS	8.61	0.63	fmol/μg protein	Choroid plexus (Plasma membrane fraction)	[9]
MATE2-k	*SLC47A2*	Targeted LC-MS/MS	ULQ < 2.19		fmol/μg protein	Choroid plexus (Plasma membrane fraction)	[9]
MATE2-K	*SLC47A2*	Targeted LC-MS/MS	ULQ < 0.295		fmol/μg protein	Brain microvessels	[8]
MCT1	*SLC16A1*	Targeted LC-MS/MS	2.27	0.85	fmol/μg protein	Brain microvessels	[8]
MCT1	*SLC16A1*	Targeted LC-MS/MS	1.87	0.22	fmol/μg protein	hCMEC/D3 (Plasma membrane fraction)	[7]
MCT1	*SLC16A1*	Targeted LC-MS/MS	5.37	3.73	pmol/mg protein	Brain microvessels	[6]
MCT1	*SLC16A1*	Targeted LC-MS/MS	3.47	0.26	fmol/μg protein	Choroid plexus (Plasma membrane fraction)	[9]
MCT10	*SLC16A10*	Targeted LC-MS/MS	ULQ < 2.6		fmol/μg protein	Choroid plexus (Plasma membrane fraction)	[9]
MCT2	*SLC16A7*	Targeted LC-MS/MS	ULQ < 0.671		fmol/μg protein	Choroid plexus (Plasma membrane fraction)	[9]
MCT2	*SLC16A7*	Targeted LC-MS/MS	ULQ < 0.277		fmol/μg protein	Brain microvessels	[8]
MCT3	*SLC16A3*	Targeted LC-MS/MS	ULQ < 0.921		fmol/μg protein	Choroid plexus (Plasma membrane fraction)	[9]
MCT4	*SLC16A4*	Targeted LC-MS/MS	0.382	0.078	fmol/μg protein	Choroid plexus (Plasma membrane fraction)	[9]
MCT5	*SLC16A5*	Targeted LC-MS/MS	0.685	0.124	fmol/μg protein	Choroid plexus (Plasma membrane fraction)	[9]
MCT8	*SLC16A2*	Targeted LC-MS/MS	1.65	0.16	fmol/μg protein	Choroid plexus (Plasma membrane fraction)	[9]
MRP4	*ABCC4*	Targeted LC-MS/MS	0.818	0.14	fmol/μg protein	Choroid plexus (Plasma membrane fraction)	[9]
NET	*SLC6A2*	Targeted LC-MS/MS	ULQ < 0.361		fmol/μg protein	Choroid plexus (Plasma membrane fraction)	[9]
NET	*SLC6A2*	Targeted LC-MS/MS	ULQ < 0.441		fmol/μg protein	Brain microvessels	[8]
NTCP	*SLC10A1*	Targeted LC-MS/MS	ULQ < 0.771		fmol/μg protein	Choroid plexus (Plasma membrane fraction)	[9]
NTCP	*SLC10A1*	Targeted LC-MS/MS	ULQ < 0.454		fmol/μg protein	Brain microvessels	[8]
OAT1	*SLC22A6*	Targeted LC-MS/MS	ULQ < 0.687		fmol/μg protein	Choroid plexus (Plasma membrane fraction)	[9]
OAT1	*SLC22A6*	Targeted LC-MS/MS	ULQ < 0.909		fmol/μg protein	Brain microvessels	[8]
OAT1	*SLC22A6*	Targeted LC-MS/MS	0.48	0.11	pmol/mg protein	Brain microvessels	[6]
OAT2	*SLC22A7*	Targeted LC-MS/MS	ULQ < 0.152		fmol/μg protein	Choroid plexus (Plasma membrane fraction)	[9]
OAT2	*SLC22A7*	Targeted LC-MS/MS	ULQ < 0.153		fmol/μg protein	Brain microvessels	[8]
OAT2	*SLC22A7*	Targeted LC-MS/MS	7.9	3.8	pmol/mg protein	Brain microvessels	[6]
OAT3	*SLC22A8*	Targeted LC-MS/MS	1.87	0.12	fmol/μg protein	Choroid plexus (Plasma membrane fraction)	[9]
OAT3	*SLC22A8*	Targeted LC-MS/MS	ULQ < 0.348		fmol/μg protein	Brain microvessels	[8]
OAT3	*SLC22A8*	Targeted LC-MS/MS	0.27	0.03	pmol/mg protein	Brain microvessels	[6]
OAT4	*SLC22A11*	Targeted LC-MS/MS	ULQ < 0.534		fmol/μg protein	Choroid plexus (Plasma membrane fraction)	[9]
OAT4	*SLC22A11*	Targeted LC-MS/MS	ULQ < 0.243		fmol/μg protein	Brain microvessels	[8]
OAT5	*SLC22A10*	Targeted LC-MS/MS	ULQ < 3.27		fmol/μg protein	Choroid plexus (Plasma membrane fraction)	[9]
OAT5	*SLC22A10*	Targeted LC-MS/MS	ULQ < 0.0898		fmol/μg protein	Brain microvessels	[8]
OAT7	*SLC22A9*	Targeted LC-MS/MS	0.51	0.1	pmol/mg protein	Brain microvessels	[6]
OATP1	*SLCO*	Targeted LC-MS/MS	0.54	0.1	pmol/mg protein	Brain microvessels	[6]
OATP1A2	*SLCO1A2*	Targeted LC-MS/MS	ULQ < 0.452		fmol/μg protein	Choroid plexus (Plasma membrane fraction)	[9]
OATP1B1	*SLCO1B1*	Targeted LC-MS/MS	ULQ < 0.303		fmol/μg protein	Choroid plexus (Plasma membrane fraction)	[9]
OATP1B3	*SLCO1B3*	Targeted LC-MS/MS	ULQ < 0.619		fmol/μg protein	Choroid plexus (Plasma membrane fraction)	[9]
OATP1C1	*SLCO1C1*	Targeted LC-MS/MS	ULQ < 0.156		fmol/μg protein	Choroid plexus (Plasma membrane fraction)	[9]
OATP1C1	*SLCO1C1*	Targeted LC-MS/MS	0.27	0.03	pmol/mg protein	Brain microvessels	[6]
OATP2B1	*SLCO2B1*	Targeted LC-MS/MS	ULQ < 0.237		fmol/μg protein	Choroid plexus (Plasma membrane fraction)	[9]
OATP2B1	*SLCO2B1*	Targeted LC-MS/MS	0.4	0.04	pmol/mg protein	Brain microvessels	[6]
OATP2B1	*SLCO2B1*	Targeted LC-MS/MS	0.48	0.11	pmol/mg protein	Brain microvessels	[6]
OATP3A1	*SLCO3A1*	Targeted LC-MS/MS	0.641	12	fmol/μg protein	Choroid plexus (Plasma membrane fraction)	[9]
OATP4A1	*SLCO4A1*	Targeted LC-MS/MS	ULQ < 1.2		fmol/μg protein	Choroid plexus (Plasma membrane fraction)	[9]
OATP4C1	*SLCO4C1*	Targeted LC-MS/MS	ULQ < 0.283		fmol/μg protein	Choroid plexus (Plasma membrane fraction)	[9]
OATP5A1	*SLCO5A1*	Targeted LC-MS/MS	ULQ < 3.28		fmol/μg protein	Choroid plexus (Plasma membrane fraction)	[9]
OATP6A1	*SLCO6A1*	Targeted LC-MS/MS	ULQ < 0.545		fmol/μg protein	Choroid plexus (Plasma membrane fraction)	[9]
OATP8	*SLCO1B3*	Targeted LC-MS/MS	0.46	0.15	pmol/mg protein	Brain microvessels	[6]
OATP-8	*SLCO1B3*	Targeted LC-MS/MS	ULQ < 0.572		fmol/μg protein	Brain microvessels	[8]
OATP-A	*SLCO1A2*	Targeted LC-MS/MS	ULQ < 0.695		fmol/μg protein	Brain microvessels	[8]
OATP-B	*SLCO2B1*	Targeted LC-MS/MS	ULQ < 0.337		fmol/μg protein	Brain microvessels	[8]
OATP-C	*SLCO1B1*	Targeted LC-MS/MS	ULQ < 0.35		fmol/μg protein	Brain microvessels	[8]
OATP-D	*SLCO3A1*	Targeted LC-MS/MS	ULQ < 0.254		fmol/μg protein	Brain microvessels	[8]
OATP-E	*SLCO4A1*	Targeted LC-MS/MS	ULQ < 0.758		fmol/μg protein	Brain microvessels	[8]
OATP-F	*SLCO1C1*	Targeted LC-MS/MS	ULQ < 0.208		fmol/μg protein	Brain microvessels	[8]
OATP-H	*SLCO4C1*	Targeted LC-MS/MS	ULQ < 0.21		fmol/μg protein	Brain microvessels	[8]
OATP-I	*SLCO*	Targeted LC-MS/MS	ULQ < 0.082		fmol/μg protein	Brain microvessels	[8]
OATP-J	*SLCO5A1*	Targeted LC-MS/MS	ULQ < 0.061		fmol/μg protein	Brain microvessels	[8]
OCTL1	*SLC22A13*	Targeted LC-MS/MS	ULQ < 0.532		fmol/μg protein	Choroid plexus (Plasma membrane fraction)	[9]
OCTL1	*SLC22A13*	Targeted LC-MS/MS	ULQ < 0.699		fmol/μg protein	Brain microvessels	[8]
OCTL2	*SLC22A14*	Targeted LC-MS/MS	ULQ < 0.698		fmol/μg protein	Choroid plexus (Plasma membrane fraction)	[9]
OCTL2	*SLC22A14*	Targeted LC-MS/MS	ULQ < 0.527		fmol/μg protein	Brain microvessels	[8]
OCTN1	*SLC22A4*	Targeted LC-MS/MS	ULQ < 0.25		fmol/μg protein	Choroid plexus (Plasma membrane fraction)	[9]
OCTN1	*SLC22A4*	Targeted LC-MS/MS	ULQ < 0.123		fmol/μg protein	Brain microvessels	[8]
OCTN1	*SLC22A4*	Targeted LC-MS/MS	ULQ < 0.04	0.01	pmol/mg protein	Brain microvessels	[6]
OCTN2	*SLC22A5*	Targeted LC-MS/MS	ULQ < 0.907		fmol/μg protein	Choroid plexus (Plasma membrane fraction)	[9]
OCTN2	*SLC22A5*	Targeted LC-MS/MS	ULQ < 0.288		fmol/μg protein	Brain microvessels	[8]
OST-α	*SLC51A*	Targeted LC-MS/MS	0.45	0.13	pmol/mg protein	Brain microvessels	[6]
PCFT	*SLC46A1*	Targeted LC-MS/MS	ULQ < 0.419		fmol/μg protein	Brain microvessels	[8]
PCFT	*SLC46A1*	Targeted LC-MS/MS	1.78	0.17	fmol/μg protein	Choroid plexus (Plasma membrane fraction)	[9]
PEPT1	*SLC15A1*	Targeted LC-MS/MS	ULQ < 0.325		fmol/μg protein	Choroid plexus (Plasma membrane fraction)	[9]
PEPT1	*SLC15A1*	Targeted LC-MS/MS	ULQ < 0.379		fmol/μg protein	Brain microvessels	[8]
PEPT2	*SLC15A2*	Targeted LC-MS/MS	ULQ < 0.216		fmol/μg protein	Brain microvessels	[8]
PEPT2	*SLC15A2*	Targeted LC-MS/MS	ULQ < 0.37		fmol/μg protein	Choroid plexus (Plasma membrane fraction)	[9]
PGT	*SLCO2A1*	Targeted LC-MS/MS	ULQ < 0.233		fmol/μg protein	Choroid plexus (Plasma membrane fraction)	[9]
PGT	*SLCO2A1*	Targeted LC-MS/MS	ULQ < 0.186		fmol/μg protein	Brain microvessels	[8]
PHT2	*SLC15A3*	Targeted LC-MS/MS	ULQ < 0.456		fmol/μg protein	Choroid plexus (Plasma membrane fraction)	[9]
PMAT	*SLC29A4*	Targeted LC-MS/MS	ULQ < 0.191		fmol/μg protein	Brain microvessels	[8]
PMAT	*SLC29A4*	Targeted LC-MS/MS	0.288	0.041	fmol/μg protein	Choroid plexus (Plasma membrane fraction)	[9]
RFC	*SLC19A*	Targeted LC-MS/MS	0.76	0.04	fmol/μg protein	Brain microvessels	[8]
RFC	*SLC19A*	Targeted LC-MS/MS	0.76	0.04	fmol/μg protein	Brain microvessels	[8]
RFC1	*SLC19A1*	Targeted LC-MS/MS	3.68	0.09	fmol/μg protein	Choroid plexus (Plasma membrane fraction)	[9]
SERT	*SLC6A4*	Targeted LC-MS/MS	ULQ < 0.304		fmol/μg protein	Choroid plexus (Plasma membrane fraction)	[9]
SERT	*SLC6A4*	Targeted LC-MS/MS	ULQ < 0.116		fmol/μg protein	Brain microvessels	[8]
SLC22A18	*SLC22A18*	Targeted LC-MS/MS	ULQ < 0.375		fmol/μg protein	Choroid plexus (Plasma membrane fraction)	[9]
SLC22A18	*SLC22A18*	Targeted LC-MS/MS	ULQ < 0.345		fmol/μg protein	Brain microvessels	[9]
TAUT	*SLC6A6*	Targeted LC-MS/MS	ULQ < 0.169		fmol/μg protein	Choroid plexus (Plasma membrane fraction)	[9]
TAUT	*SLC6A6*	Targeted LC-MS/MS	ULQ < 0.0767		fmol/μg protein	Brain microvessels	[8]
TfR1	*TFRC*	Targeted LC-MS/MS	2.34	0.76	fmol/μg protein	Brain microvessels	[8]
URAT1	*SLC22A12*	Targeted LC-MS/MS	ULQ < 0.357		fmol/μg protein	Choroid plexus (Plasma membrane fraction)	[9]
URAT1	*SLC22A12*	Targeted LC-MS/MS	ULQ < 0.0566		fmol/μg protein	Brain microvessels	[8]
UST3	*SLC22A9*	Targeted LC-MS/MS	ULQ < 1.21		fmol/μg protein	Choroid plexus (Plasma membrane fraction)	[9]
UST3	*SLC22A9*	Targeted LC-MS/MS	ULQ < 0.326		fmol/μg protein	Brain microvessels	[8]
xCT	*SLC7A11*	Targeted LC-MS/MS	ULQ < 0.783		fmol/μg protein	Choroid plexus (Plasma membrane fraction)	[9]
xCT	*SLC7A11*	Targeted LC-MS/MS	ULQ < 0.429		fmol/μg protein	Brain microvessels	[8]

A summary of the uptake transporters proteomics-based absolute abundance (fmol/μg protein or pmol/mg protein) in human brain microvascular cells or choroid plexus. Data showing individual proteomics expression profiles of uptake transporters in the available literature.

**Table 2:** Species differences in uptake drug transporters involved in brain drug exposure. A summary of the literature-reported brain drug exposure in different species.

Species	Transporter	Substrate	Perpetrator	Method	Exposure	DDI/Effect	Expression Level (fmol/μg Protein)	Reference
Mouse	Oatp1a4	Glyburide	Rifampicin	In situ brain perfusion	Kin (0.5 ± 0.11 μL/g/s)	No change (Kin: ~0.4)		[87]
Mouse	Oatp1a4	Rosuvastatin	-	In vivo brain uptake	57 ± 9 μL/min/g brain (brain/plasma = 22 μL/g)	-		[88]
		Pravastatin			24 ± 4 μL/min/g brain (brain/plasma = 8.3 μL/g)			
		Taurocholate			11 ± 2 μL/min/g brain			
		Ochratoxin A			11 ± 0 μL/min/g brain (brain/plasma = 7.6 μL/g)			
Mouse (Bend.3)	OCT1, OCT2, OCT3	Pentamidine	Amantadine (500 μM)	In vitro uptake (V_d_)	V_d_ at different time points	59% Reduction		[96]
			Prazosin (100 μM)			No change (paracellular leakage increased)		
			N-methy-l nicotinamide (100 μM)			No change		
Rat	Oatp1a4	-	-		-	-	1.99	[49]
Rat	Oct1/2	Memantine (5 mh/kg i.v.)	Cimetidine (25 μM)	In vivo brain uptake	84.59 ± 9.73 pmol/mg brain tissue	37% Decreased (54.14 ± 8.35)	-	[82]
Rat	Oatp1a4	Atorvastatin	BMP-9 (1 μg/kg)	In vivo brain uptake	AUC: 987.9 ± 53.41 pmol × min/mg brain tissue	60% Increased (1581 ± 52.26)	-	[83]
			LDN (10 mg/kg) + BMP-9			Attenuated the BMP-9 effect		
		Pravastatin	BMP-9 (1 μg/kg)		AUC: 800.0 ± 47.41 pmol × min/mg brain tissue	69% Increased (1349.00 ± 48.00)		
			LDN (10 mg/kg) + BMP-9			Attenuated the BMP-9 effect		
		Rosuvastatin	BMP-9 (1 μg/kg)		AUC: 836.8 ± 50.53 pmol × min/mg brain tissue	74% Increased (1459.0 ± 53.51)		
			LDN (10 mg/kg) + BMP-9			Attenuated the BMP-9 effect		
Rat	Oatp1a4	Atorvastatin	Fexofenadine (100 μM)	In situ brain perfusion	63.72 ± 9.78 pmol/mg brain tissue	39% reduced (24.89 ± 7.55)	-	
		Pravastatin	Fexofenadine (100 μM)		54.98 ± 6.37 pmol/mg brain tissue	Reduced (12.39 ± 4.8)		
		Rosuvastatin	Fexofenadine (100 μM)		55.83 ± 7.84 pmol/mg brain tissue	Reduce (10.54 ± 3.65)		
Rat	Octs	SHY-01 (50 mg/kg)	-	In vivo	2.05 ± 0.18 (hr·μg/mL)	CL: 24.48 ± 2.25	-	[84]
		Metformin (50 mg/kg)	-	In vivo	1.89 ± 0.08 (hr·μg/mL)	CL: 26.46 ± 1.10		
Rat	Oatp	Digoxin (2 mg/kg, i.v.)	Rifampicin (30 mg/kg, oral)	In vivo	~0.07 (K_p,AUC,brain_)	Increased (~1.8-fold)	-	[85]
					~0.02 (K_p,AUC,CSF_)	Increased (~4-fold)		
Rat	Oatp1a4	Taurocholate	BMP-9 (1 μg/kg)	In vivo brain uptake	AUC: 1143.6 ± 57.92 pmol × min/mg brain tissue)	79% Increased (2054.83 ± 66.13)	-	[89]
			E3S (100 μM)	In situ brain perfusion	65.31 ± 8.19 pmol/mg	59% Reduced (27.02 ± 7.56)		
			Fexofenadine (100 μM)			61% Reduced (25.61 ± 7.44)		
			BSP			No effect (66.81 ± 7.13)		
		Atorvastatin	E3S (100 μM)		34.07 ± 5.67 pmol/mg brain tissue	Reduced (17.67 ± 5.22 pmol/mg brain tissue)		
		Pravastatin			22.01 ± 6.27 pmol/mg brain tissue	Reduced (9.00 ± 4.98 pmol/mg brain tissue)		
Rat	Oatp1a4	Taurocholate	E3S (100 μM)	In situ brain perfusion	~55 pmol/g brain tissue	Reduced (2.2-fold)	-	[94]
			Digoxin (200 μM)			Reduced (2.4-fold)		
			Fexofenadine (100 μM)		V_brain_ = 97.61 ± pmol/g	Reduced (2.2-fold)		
			BSP			No effect		
Rat	Oats, Mrps, Oatps	Cefadroxil	Probenecid	Microdialysis	AUC_blood_ = 1802 ± 97 (μg × min/mL)	2873 ± 177 (Increased)	-	[95,97]
					AUC_ECF_ = 40 ± 7	174 ± 35 (Increased)		
					Kp,_uu,ECF_ = 0.022 ± 0.003	0.058 ± 0.009 (Increased)		
					AUC_CSF_ = 57 ± 15	117 ± 50 (Increased)		
					Kp_,uu,CSF_ = 0.031 ± 0.007	0.039 ± 0.015		
	Pept2	Cefadroxil	Ala-Ala	Brain slices	V,_u,brain_ (mL/g brain) = 3.67 ± 0.23	0.95 ± 0.45 (Reduced)		
			GlySar			1.10 ± 0.05 mL/g (Reduced)		
	Oats, Mrps, Oatps		Probenecid			6.06 ± 0.15 (Increased)		
Dog	OCT2		-	LC-MS/MS	-	-	<LOQ	[90,92,93]
		-	-		-	-	<LOQ	
	OAT3	-	-		-	-	<LOQ	
		-	-		-	-	<LOQ	
	OATP1A2	-	-		-	-	<LOQ	
		-	-		-	-	2.69 ± 0.78	
	OATP2B1	-	-		-	-	<LOQ	
		-	-		-	-	<LOQ	
	ENT1	-	-		-	-	0.581 ± 0.342	
		-	-		-	-	1.05 ± 0.47	
	LAT1	-	-		-	-	<LOQ	
		-	-		-	-	<LOQ	
	OCT3/P-gp	Quinidine (7.71 μmol/kg)	-	In vivo brain uptake	K,_p,uu,brain_ = 0.363 ± 0.11	-	-	
			-		K,p,uu,csf = 0.131 ± 0.036	-		
	OAT2/BCRP	Dantrolene (1.59 μmol/kg)	-		Kp,uu,brain = 0.0614 ± 0.0021	-	-	
			-		K,p,uu,csf = 0.505 ± 0.025	-		
Monkey (Baboon)	OATP2B1, OATP1A2	Glyburide	Rifampicin	PET	4.5 ± 1.0 (AUC_brain_/AUC_blood_ = 0.032)	No change: 11.5 (0.018)	-	[87]
			Cyclosporine			No change: 17.2 (0.029)		
			Pantoprazole			No change: 8.1 (0.035)		
Monkey	OATP2B1	-	-		-	-	0.12	[49]
Human (hCMEC/D3)	OCT1, OCT2, OCT3	Pentamidine	Amantadine (500 μM)	In vitro uptake (V_d_)	V_d_ at different time points	45% Reduction	-	[96]
			Prazosin (100 μM)			39% Reduction		
			N-methy-l nicotinamide (100 μM)			No change		
Human	OATP2B1, OATP1A2	Glyburide	Rifampicin (9 mg/kg i.v.)	PET	5.82 ± 0.74 (AUC_brain_/AUC_blood_ = 0.03)	No change: 7.72 (0.03)	-	[86]

A summary of the literature-reported brain drug exposure in rodent models. The data show brain exposure, tissue partition coefficient (*_kp,uu_*), and transporter-mediated drug–drug interaction on brain concentrations of these uptake and/or efflux transporter substrate drugs.

**Table 3:** Physicochemical properties and transporter interactions in brain disposition of therapeutic drugs.

Drug Name	Drug Class	Mw ^a^	LogD ^b^	Plasma Protein Binding (%) ^a^	Efflux Transporter Substrate		Uptake Transporter Substrate														*K*_*p*,*uu*,*brain*_			
					MDR1	BCRP	OATP1B1	OATP1B3	OATP2B1	OATP1A2	OCT1	OCT2	OCT3	OCTN1	OCTN2	LAT1	OAT1	OAT3	MATE1	MATE2k	Rat	Mouse	Monkey	Human
**Dolutegravir**	HIV-Integrase strand transfer inhibitor	419.38	1.10	98.90	Yes [98]	Yes [98]	NA	NA	NA	NA	NA	NA	NA	NA	NA	NA	NA	NA	NA	NA	0.02 [99]	NA	NA	NA
**Efavirenz**	Non-nucleoside Reverse transcriptase inhibitors (NNRTI)	315.68	4.46	99.60	No [100]	NA	No [101]	No [101]	NA	No [101]	No [102]	No [102]	NA	NA	NA	NA	NA	NA	NA	NA	0.20 [103]	NA	NA	NA
**Erlotinib**	Kinase inhibitor	393.40	3.05	93.00	Yes [104]	Yes [104]	No [105]	No [105]	Yes [54]	NA	No [106]	Yes [106]	NA	NA	NA	NA	No [106]	Yes [106]	NA	NA	0.06 [104]	NA	0.05 [104]	0.08 [107]
**Fexofenadine**	H-1 Receptor antagonists	501.66	2.93	65.00	Yes [108]	No [109]	Yes [110]	Yes [111]	Yes [112]	Yes [113]	Yes [114]	No [115]	No [112]	NA	No [112]	NA	Yes [116]	Yes [116]	Yes [117]	Yes [117]	0.05 [118]	0.22 [26]	NA	NA
**Gabapentin**	Anticonvulsant	171.20	−1.27	<3	No [119]	NA	NA	NA	NA	No [120]	No [121]	Yes [122]	No [123]	Yes [124]	No [121]	Yes [122]	No [122]	No [122]	NA	NA	0.14 [125]	NA	NA	0.16 [125]
**Lamotrigine**	Anticonvulsants	256.10	1.91	55.00	No [104]	No [104]	NA	NA	NA	No [120]	Yes [126]	Yes [126]	Yes [126]	No [127]	No [127]	NA	NA	NA	NA	NA	0.88 [125]	NA	0.86 [104]	2.80 [128]
**Loperamide**	Antidiarrheal	477.10	2.77	95.00	Yes [104]	No [104]	NA	NA	NA	NA	NA	NA	NA	NA	NA	NA	NA	NA	NA	NA	0.02 [128]	NA	0.04 [129]	NA
**Methotrexate**	Antimetabolite	454.40	−6.56	50.25	Yes [130]	Yes [131]	Yes [132]	Yes [133]	Yes [134]	Yes [134]	NA	Yes [135]	NA	NA	NA	NA	Yes [136]	Yes [137]	Yes [135]	Yes [135]	0.006 [125]	NA	0.04 [104]	NA
**Pitavastatin**	HMG CoA Reductase Inhibitors (statin)	421.50	0.89	>99	Yes [104]	Yes [104]	Yes [138]	Yes [139]	Yes [140]	Yes [141]	No	NA	NA	NA	NA	NA	NA	NA	NA	NA	NA	NA	0.24 [104]	NA
**Quinidine**	Antiarrhythmic	324.40	0.86	78.00	Yes [104]	No [104]	NA	NA	NA	NA	NA	NA	Yes [92]	Yes [142]	No [143]	NA	NA	NA	No [144]	No [144]	0.04 [104]	NA	0.10 [104]	NA
**Raltegravir**	HIV-Integrase Strand transfer inhibitor	444.42	−0.92	83.00	Yes [5]	Yes [5]	No [145]	No [145]	NA	No [145]	No [145]	NA	NA	NA	No [145]	NA	Yes [146]	NA	NA	NA	0.13 [147]	NA	0.12 [147]	NA
**Rifampicin**	Antibiotic	822.90	2.87	89.00	Yes [148]	No [149]	Yes [150]	Yes [150]	No [150]	No [150]	No [102]	No [102]	No [151]	NA	NA	NA	NA	NA	NA	NA	0.04 [125]	NA	NA	NA
**Rosuvastatin**	HMG CoA Reductase Inhibitors (statin)	481.54	−1.24	88.00	Yes [152]	Yes [153]	Yes [154]	Yes [154]	Yes [155]	Yes [156]	No [139]	NA	NA	NA	NA	NA	NA	NA	NA	NA	3.97 [157]	NA	NA	NA
**Zidovudine**	Nucleoside Reverse Transcriptase Inhibitors (NRTI)	267.20	−0.41	<38	Yes [104]	Yes [104]	NA	NA	NA	NA	No [158]	No [158]	No [159]	NA	NA	NA	Yes [160]	Yes [158]	NA	NA	0.09 [125]	NA	NA	NA

Data show potential interactions of drugs with efflux and uptake transporters and the effect on brain exposure. We summarized their reported Kp,uu,brain in rodents, monkey, and human to justify the role of the active transport in their brain disposition via BBB. ^a^ Retrieved information from DrugBank database. ^b^ Retrieved information from ChEMBL database. NA: Not available.

## Data Availability

All raw data was extracted from the literature that will be available upon request.

## Abbreviations

CNTs: concentrative nucleoside transporters; ENTs: equilibrative nucleoside transporters; MCTs: monocarboxylate transporters; OATPs: organic anion transporter polypeptides; PMAT: plasma membrane monoamine transporter; LATs: L-type amino acid transporters; OATs: organic anion transporters; OCTs: organic cation transporters; MATE: multidrug and toxic compound extrusion; PEPTs: peptide transporters; 4F2hc: several heterodimeric amino acids transporter; ASBT: apical sodium-dependent bile acid transporter; ASCT: alanine serine cysteine transporter; ATA: amino acid transporter; BGT: gamma-aminobutyric acid transporter; BOCT: brain organic cation transporter; CRT: creatine transporter; CT: human l-carnitine transporter; CTL: choline transporter; EAAT: excitatory amino acid transporter; FATP: long-chain fatty acid transport protein; GAT: gamma-aminobutyric acid (GABA) transporter; GLUT: glucose transporters; NET: norepinephrine transporter; OSTα: organic solute transporter alpha; PCFT: proton-coupled folate transporter; PGT: prostaglandin transporter; PHT2: peptide histidine transporter 2; RFC: reduced folate transporter; SERT: serotonin transporter; TAUT: sodium- and chloride-dependent taurine transporter; TfR1: transferrin receptor 1; URAT: urate transporter; UST: sodium-independent organic anion transporter; xCT: cystine/glutamate antiporter. ULQ represents the limit of quantification. In case of no positive peak or just one or two SRM/MRM transitions for a specific protein, the protein expression was determined as ULQ.
